# Disabling the Protease DDI2 Attenuates the Transcriptional Activity of NRF1 and Potentiates Proteasome Inhibitor Cytotoxicity

**DOI:** 10.3390/ijms21010327

**Published:** 2020-01-03

**Authors:** Amy Northrop, Janakiram R. Vangala, Alex Feygin, Senthil K. Radhakrishnan

**Affiliations:** Department of Pathology and Massey Cancer Center, Virginia Commonwealth University, Richmond, VA 23298, USA; northropav@mymail.vcu.edu (A.N.); janakiram.vangala@vcuhealth.org (J.R.V.); alex.feygin@vcuhealth.org (A.F.)

**Keywords:** NRF1, DDI2, proteasome genes, transcription, proteasome inhibitor, cancer

## Abstract

Proteasome inhibition is used therapeutically to induce proteotoxic stress and trigger apoptosis in cancer cells that are highly dependent on the proteasome. As a mechanism of resistance, inhibition of the cellular proteasome induces the synthesis of new, uninhibited proteasomes to restore proteasome activity and relieve proteotoxic stress in the cell, thus evading apoptosis. This evolutionarily conserved compensatory mechanism is referred to as the proteasome-bounce back response and is orchestrated in mammalian cells by nuclear factor erythroid derived 2-related factor 1 (NRF1), a transcription factor and master regulator of proteasome subunit genes. Upon synthesis, NRF1 is cotranslationally inserted into the endoplasmic reticulum (ER), then is rapidly retrotranslocated into the cytosol and degraded by the proteasome. In contrast, during conditions of proteasome inhibition or insufficiency, NRF1 escapes degradation, is proteolytically cleaved by the aspartyl protease DNA damage inducible 1 homolog 2 (DDI2) to its active form, and enters the nucleus as an active transcription factor. Despite these insights, the cellular compartment where the proteolytic processing step occurs remains unclear. Here we further probed this pathway and found that NRF1 can be completely retrotranslocated into the cytosol where it is then cleaved and activated by DDI2. Furthermore, using a triple-negative breast cancer cell line MDA-MB-231, we investigated the therapeutic utility of attenuating DDI2 function. We found that DDI2 depletion attenuated NRF1 activation and potentiated the cytotoxic effects of the proteasome inhibitor carfilzomib. More importantly, expression of a point-mutant of DDI2 that is protease-dead recapitulated these effects. Taken together, our results provide a strong rationale for a combinational therapy that utilizes inhibition of the proteasome and the protease function of DDI2. This approach could expand the repertoire of cancer types that can be successfully treated with proteasome inhibitors in the clinic.

## 1. Introduction

In eukaryotes, the majority of intracellular proteins are degraded via the ubiquitin-proteasome pathway (UPP). The selective poly-ubiquitination of degradation-targeted proteins is coupled with recognition by the proteasome followed by proteolysis within its catalytic core. The UPP is tightly regulated to maintain proper protein homeostasis, or proteostasis, which is critical to cellular health and survival [1,2]. A build-up of ubiquitinated proteins creates proteotoxic stress and can trigger an apoptotic cellular response, thus is the basis for proteasome inhibitors (PIs) as cancer therapy [3,4,5].

To date, three PIs have gained Food and Drug Administration (FDA) approval. Bortezomib (BTZ) is currently approved as a first-line therapy for multiple myeloma and mantle cell lymphoma, while Carfilzomib (CFZ) and Ixazomib (IXZ) are approved for relapsed and refractory multiple myeloma (RRMM). In general, hematologic tumors cells have proven more sensitive to PI-induced apoptosis than solid tumors [6,7,8]. The major difference between these PIs, aside from their route of delivery (intravenous for BTZ and CFZ; oral for IXZ), is the mechanism by which they inhibit the proteasome. BTZ and IXZ reversibly bind the target catalytic site within the core of the proteasome, whereas CFZ binds irreversibly [4].

The binding mechanism of the PIs is especially important when considering the evolutionarily well-conserved proteasome bounce-back response that is orchestrated in response to proteasome inhibition. The bounce-back response is an upregulation of proteasome subunit (PSM) gene expression upon inhibition of the proteasome, leading to the recovery of proteasome activity in the cell [9,10,11,12]. With the use of reversible PIs, the cell has two options for overcoming the inhibition—wait for the PI to release, allowing the previously inhibited proteasome to resume proteolytic activity, or activate the bounce-back response to produce new proteasomes. In the case of irreversible inhibition, the cell’s only option is to utilize the bounce-back response [12].

We and others determined the mediator of the proteasome bounce-back response to be the transcription factor nuclear factor erythroid-derived 2-related factor 1 (NRF1) in mammalian cells [12,13], comparable to the transcription factors RPN4 in yeast [14] and Cnc-C in Drosophila [15]. NRF1 binds the anti-oxidant response elements (ARE) found in the promoter region of PSM genes [16] and the genetic ablation or knockdown of NRF1 in mouse embryonic fibroblasts results in a loss of PSM gene upregulation following proteasome inhibition [12]. Our work also demonstrated that cancer cells can be sensitized to PI-induced apoptosis when NRF1 is depleted [12], thereby establishing the notion that attenuation of the bounce-back response could improve the efficacy of PI-mediated cancer therapy. Thus, further characterization of the NRF1-dependent bounce-back response via a more complete understanding of the molecular players involved could offer novel strategies to target this pathway.

In unperturbed cellular conditions, NRF1 is maintained at low basal levels via endoplasmic reticulum (ER)-associated degradation (ERAD). In this pathway, NRF1 is co-translationally inserted into the ER membrane by a sec61-dependent mechanism [13,17], glycosylated in the C-terminal ER-embedded region [17,18], retrotranslocated out of the ER and into the cytosol by the ATPase p97/VCP, deglycosylated by the p97-interacting glycanase NGLY1 [13,17,18], ubiquitinated by the E3 ligase HRD1 [13,19], and degraded by the proteasome.

During proteasome inhibition or times of decreased proteasome activity, NRF1 is put through an activation pathway that culminates in the active NRF1 transcription factor that induces the bounce-back response to increase proteasome activity. In this activation pathway, instead of being degraded by the proteasome, NRF1 is cleaved to its active form by the protease DNA damage inducible 1 homolog 2 (DDI2) [20], then translocated into the nucleus as an active transcription factor to associate with co-factors such as small Maf proteins [21] and TIP60 [22] to increase PSM gene expression. Likewise, SKN1A, the *Caenorhabditis elegans* ortholog of NRF1 is proteolytically processed and activated by DDI1 [23]. It has been shown that genetic or chemical inhibition of p97 [13,17], NGLY1 [18], HRD1 [13], TIP60 [22], or DDI2 [20] impedes the activation of NRF1. Notably, chemical inhibition of NGLY1 in chronic myelogenous leukemia and cervical cancer cells [18] or p97 in multiple myeloma cells [24] potentiated the apoptotic effect of proteasome inhibition, further strengthening the hypothesis that crippling the bounce-back response can increase the efficacy of PIs as cancer therapy.

To date, it has not been demonstrated if impairing DDI2 can sensitize cancer cells to proteasome inhibitor-induced apoptosis. As there is no known inhibitor of DDI2 at this time, here we employed genetic tools to evaluate DDI2 as a therapeutic target in combination with proteasome inhibition. We have confirmed that DDI2 is critical to the activation of the NRF1-mediated bounce-back response, refined the model of DDI2-mediated proteolytic processing of NRF1, and demonstrated increased sensitivity of DDI2-deficient and protease-dead DDI2-expressing breast cancer cells to CFZ-induced apoptosis.

## 2. Results

### 2.1. DDI2 Is Required for NRF1-Mediated Proteasome Bounce-Back Response

DDI2 was recently identified as a protease that cleaves and activates NRF1 [20]. To further characterize the role of DDI2 in the NRF1 pathway, we engineered a DDI2-knockout NIH-3T3 mouse fibroblast cell line using the CRISPR/Cas9 method [25]. In parallel, we also generated a control NIH-3T3 cell line that expresses an EGFP-targeting gRNA. We chose NIH-3T3 cell line for the initial mechanistic studies because in mouse cells, NRF1 migrates as discrete p120 (precursor) and p110 (proteolytically-processed active form) bands in immunoblots, thus making the interpretations clearer. This is in contrast to human cells, wherein the additional presence of TCF11, an isoform of NRF1 with an extra 30 amino acids, complicates visualization of the p120 and p110 bands by western blot [13].

**Figure 1:**
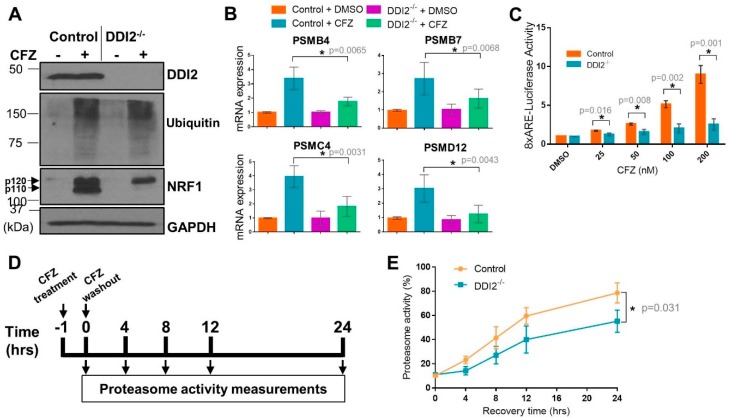
DDI2 is required for the proteolytic processing and transcriptional activity of nuclear factor erythroid derived 2-related factor 1 (NRF1). (**A**) NIH-3T3 control (expressing EGFP sgRNA) and DDI2^−/−^ cells were treated with 200 nM carfilzomib (CFZ) or equal volume DMSO for 4 h, and then analyzed by western blot using the antibodies indicated. GAPDH was used as a loading control. Blots shown are representative of three independent experiments. (**B**) NIH-3T3 control (expressing EGFP sgRNA) and DDI2^−/−^ cells were treated with 200 nM CFZ or equal volume DMSO for 8 h. RNA extracted from the cells was converted to cDNA, and then used for quantitative RT-PCR with primers for the genes indicated. mRNA levels of *GAPDH* were used for normalization. Error bars denote standard deviation (*n* = 5 for *PSMB4* and *n* = 6 for *PSMB7*, *PSMC4,* and *PSMD12*). (**C**) NIH-3T3 control (expressing EGFP sgRNA) and DDI2^−/−^ cells were transfected with a construct containing 8 consecutive antioxidant response elements (8xARE (anti-oxidant response elements)) in a promoter region upstream of a firefly luciferase gene to measure NRF1 transcriptional activity and a human phosphoglycerate kinase promoter (ubiquitously expressed) upstream of renilla luciferase gene for normalization. At 48 h post-transfection, cells were treated with DMSO or indicated concentrations of CFZ overnight, after which dual luciferase assays were used to measure firefly and renilla luciferase activity. Normalized luciferase activity is shown. Error bars denote standard deviation (*n* = 3). (**D**) Schematic of the proteasome recovery assay. (**E**) NIH-3T3 control (expressing EGFP sgRNA) and DDI2^−/−^ cells were treated with 50 nM CFZ for an hour, after which the drug was washed out with PBS and cells were allowed to recover in normal media. Proteasome activity in the lysates was measured at the indicated time points. The results were normalized to the DMSO-treated control. Error bars denote standard deviation (*n* = 3). Significance (*p* value < 0.05) is denoted by asterisk.

Both control and DDI2^−/−^ NIH-3T3 cells showed extensive accumulation of ubiquitinated proteins in response to carfilzomib (CFZ), as expected due to proteasome inhibition (Figure 1A). Under these conditions, while control cells showed accumulation of both p120 and p110 forms of NRF1 after CFZ treatment, DDI2^−/−^ cells displayed accumulation of the p120 form alone (Figure 1A), consistent with the requirement for DDI2 in proteolytically generating the p110 form. RT-qPCR of the control and DDI2^−/−^ cell lines also showed an attenuation of transcriptional bounce-back response for four of NRF1′s target proteasome subunit (PSM) genes, *PSMB4*, *PSMB7*, *PSMC4*, and *PSMD12*, in the DDI2^−/−^ cells after CFZ treatment (Figure 1B). In an orthogonal experiment, an NRF1-responsive luciferase reporter (8xARE-Luc construct characterized in [22]) displayed a dose-dependent increase in promoter-luciferase activity in response to CFZ in the control, but not in DDI2^−/−^ cells (Figure 1C). As components of the 20S proteasome core complex have been reported to have a half-life of 192 h [26], western blotting for proteasome subunit expression changes would require prolonged proteasome inhibitor treatment, which is usually associated with significant cell death that may confound the results, thus precluding the analysis of protein levels of the proteasome subunits.

Next, in order to characterize the bounce-back response defect in the DDI2^−/−^ cells at a functional level, we performed a proteasome recovery assay that we have optimized in our previous studies [12,22]. In this assay, we treated control and DDI2^−/−^ cells with a dose of CFZ sufficient to inhibit proteasome activity by ~90% (compared to DMSO-treated cells), then followed the recovery of proteasome activity in these cells for up to 24 h after drug washout (Figure 1D). DDI2^−/−^ cells were significantly impaired in their ability to recover proteasome activity when compared to control cells (Figure 1E). Taken together, our data reinforce a critical role for DDI2 in the NRF1-mediated proteasome bounce-back response in the aftermath of proteasome inhibition.

### 2.2. NRF1 Is Proteolytically Processed by DDI2 in the Cytosol

**Figure 2:**
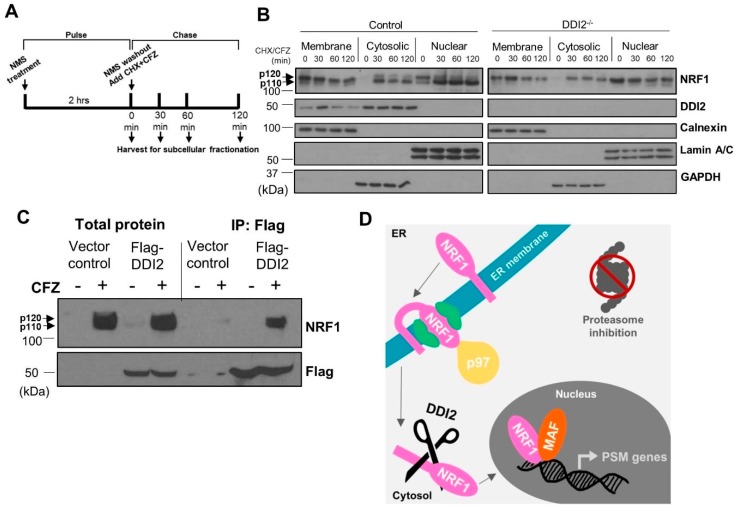
DDI2-mediated proteolytic processing of NRF1 occurs in the cytosol. (**A**) Schematic representation of the pulse-chase assay is shown. (**B**) NIH-3T3 control (wild-type) and DDI2^−/−^ cells were pulsed with 10 μM NMS-873 (p97 inhibitor) for 2 h, then chased with 50 μg/mL cycloheximide (CHX) and CFZ (5 μM) for 0, 30, 60, or 120 min. Subcellular fractionation of membrane-bound, cytosolic, and nuclear proteins was analyzed by western blot using the antibodies indicated. Calnexin, Lamin A/C, and GAPDH are fractionation controls for membrane-bound, nuclear, and cytosolic proteins, respectively. Blots shown are representative of three independent experiments. (**C**) MDA-MB-231 cells stably expressing Flag-DDI2 or a vector control were treated with 200 nM carfilzomib (CFZ) or equal volume DMSO for 4 h. The lysates derived from these cells were then subjected to immunoprecipitation with anti-Flag affinity gel. Input and immunoprecipitated proteins were probed for by western blot using the indicated antibodies. Blots shown are representative of three independent experiments. (**D**) A proposed model of NRF1 activation by DDI2 is shown. When the proteasome is inhibited, NRF1 is extracted out of the ER membrane via the action of ATPase p97 into the cytosol. NRF1 is then cleaved by DDI2 before it translocates into the nucleus as an active transcription factor.

During translation, p120 NRF1 protein is inserted into the ER membrane in an N_cytosol_–C_lumen_ orientation [27], such that only a small portion of its N-terminus protrudes into the cytosol. Other segments, including NRF1′s DNA-binding and transcriptional activation domains, remain embedded in the ER lumen. Our previous study demonstrated that the action of the ATPase p97/VCP is required to re-position the C-terminus of NRF1 into the cytosol via retrotranslocation, which facilitates its proteolytic processing and release of the NRF1 active fragment p110 [17]. To better understand the spatiotemporal aspects of NRF1 cleavage, we performed pulse-chase experiments followed by subcellular fractionation. Here, the cells were pulsed for two hours with a p97 inhibitor, NMS-873 [28], to prevent NRF1 retrotranslocation out of the ER and cause accumulation of the unprocessed p120 form. NMS-873 was then washed out and the cells were exposed to CFZ and cycloheximide for the indicated time points (Figure 2A). CFZ inhibits degradation of NRF1, while cycloheximide inhibits new protein translation, allowing us to track a single pool of NRF1 as it gets processed into the p110 form and traverses into the nucleus.

Subcellular fractionation to isolate membrane-bound, cytosolic, and nuclear proteins followed by western blot revealed that NRF1, regardless of cleavage by DDI2, is retrotranslocated out of the ER membrane and into the cytosol after NMS-873 washout (Figure 2B). Our results also indicate that NRF1 is able to enter the nucleus without cleavage (Figure 2B), though not as an active transcription factor (Figure 1B,C). Surprisingly, we also observed significant levels of nuclear NRF1 immediately after NMS-873 washout (time = 0 min in nuclear fractions in Figure 2B). We speculate that this nuclear pool of NRF1 could actually be a p97 substrate usually targeted for degradation and, hence, accumulates in our case after inhibition of p97 by NMS-873.

The study that identified DDI2 as the NRF1-activating protease did so using genetic ablation and manipulation techniques [20]. Here, we used immunoprecipitation followed by western blot to show that immunoprecipitation of flag-tagged DDI2 also pulls down NRF1, demonstrating an interaction between the proteins (Figure 2C). Taken together, our results are consistent with a model in which p120 NRF1 is completely pulled out into the cytosol, where it is cleaved by DDI2 to the active p110 form (Figure 2D).

### 2.3. DDI2-Deficient MDA-MB-231 Cells Are More Sensitive to CFZ-Induced Apoptosis

**Figure 3:**
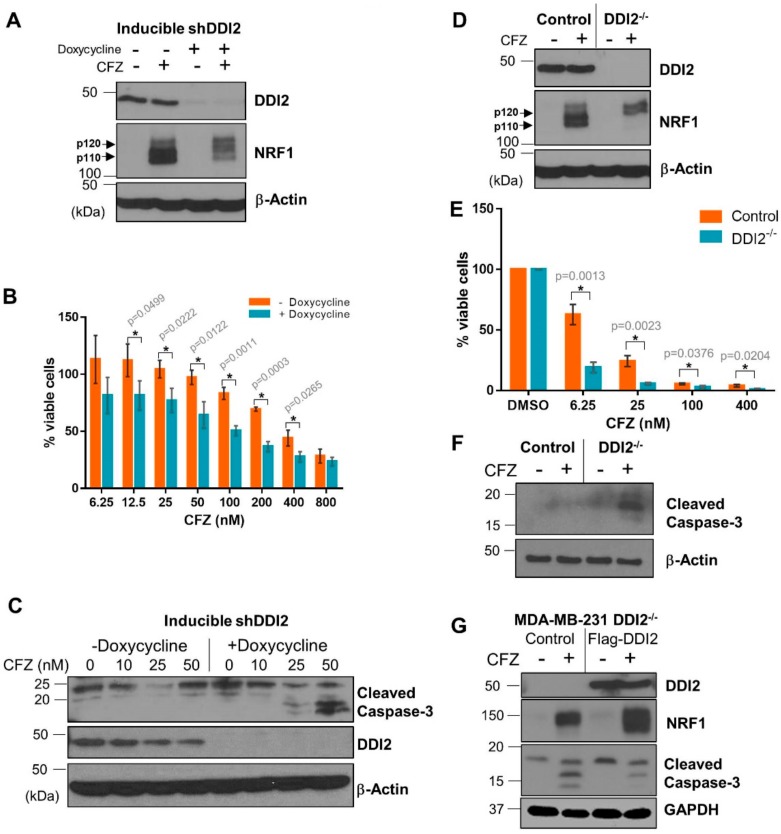
DDI2-deficient MDA-MB-231 cells are more sensitive to carfilzomib-induced cell death. (**A**) MDA-MB-231 cells stably expressing a doxycycline-inducible DDI2 shRNA construct (inducible shDDI2 cells) were incubated with or without doxycycline for ≥72 h, then were treated with 200 nM CFZ or equal volume DMSO for 4 h and analyzed by western blot using the antibodies indicated. β-Actin was used as a loading control. Blots shown are representative of three independent experiments. (**B**) MDA-MB-231 inducible shDDI2 cells were treated with or without doxycycline for ≥72 h, then were treated with increasing doses of CFZ (6.25–800 nM) for 24 h and analyzed by a luminescent cell viability assay. Viability percentage was determined via comparison to viability of untreated cells (100%). Error bars denote standard deviation (*n* = 3). (**C**) MDA-MB-231 inducible shDDI2 cells were treated with or without doxycycline for ≥72 h, then were treated with DMSO or 10, 25, or 50 nM CFZ for 24 h and analyzed by western blot using the antibodies indicated. β-Actin was used as a loading control. Blots shown are representative of three independent experiments. (**D**) MDA-MB-231 control (expressing EGFP sgRNA) and DDI2^−/−^ cells were treated with 200 nM CFZ or equal volume DMSO for 4 h and analyzed by western blots using the indicated antibodies. β-Actin was used as a loading control. Blots shown are representative of three independent experiments. (**E**) MDA-MB-231 control (expressing EGFP sgRNA) and DDI2^−/−^ cells were treated with DMSO or increasing doses of CFZ (6.25–400 nM) for 24 h and analyzed by MTT assay. Viability percentage was determined via comparison to viability of DMSO-treated cells (100%). Error bars denote standard deviation (*n* = 3). (**F**) MDA-MB-231 control (expressing EGFP sgRNA) and DDI2^−/−^ cells were treated with 10 nM CFZ or equal volume DMSO for 24 h, then the cell lysates were analyzed by western blot with the indicated antibodies. β-Actin was used as a loading control. Blots shown are representative of three independent experiments. (**G**) MDA-MB-231 DDI2^−/−^ cells expressing a vector control or CRISPR-resistant Flag-WT-DDI2 were treated with 20 nM CFZ or DMSO for 24 h and the cell lysates were subjected to immunoblotting with indicated antibodies. GAPDH was used as a loading control. Blots shown are representative of three independent experiments. Significance (*p* value < 0.05) is denoted by asterisk.

Solid tumors, like breast cancer, have been historically less sensitive to treatment with proteasome inhibitors [7,8]. We reasoned that DDI2 depletion could sensitize these cancer cells to CFZ-induced apoptosis via inhibition of the NRF1-mediated bounce-back response. To test this hypothesis, we first generated a triple negative breast cancer (TNBC) cell line MDA-MB-231 with stable expression of doxycycline-inducible DDI2 shRNA. These cells, when incubated with doxycycline for ≥72 hours showed a robust knockdown of DDI2 and displayed deficient NRF1 processing in response to CFZ treatment (Figure 3A). More importantly, in an MTT cell viability assay, DDI2-depleted cells showed significantly decreased viability across various CFZ concentrations compared to the control cells (Figure 3B). Also, these DDI2-depleted MDA-MB-231 cells showed enhanced levels of cleaved caspase-3 (a marker for apoptosis) following treatment with CFZ when compared to control (Figure 3C). To independently confirm these results in an orthogonal model, we generated MDA-MB-231 DDI2^−/−^ cells using CRISPR/Cas9. These DDI2^−/−^ cells recapitulated all of the observations that were made in the inducible knockdown system–deficient NRF1 processing and increased sensitivity to CFZ, as measured by MTT assays and cleaved caspase-3 levels (Figure 3D–F). To confirm that the increased sensitivity was due to specific depletion of DDI2, we rescued DDI2 expression by introducing a CRISPR-resistant Flag-WT-DDI2 expression construct into the DDI2^−/−^ cells. Expression of CRISPR-resistant Flag-DDI2 led to a rescue in NRF1 processing and decreased cleaved caspase-3 levels following treatment with CFZ (Figure 3G).

### 2.4. DDI2-Deficient MDA-MB-231 Cells Are More Sensitive to CFZ-Induced Apoptosis in an In Vitro Model of Rapid Clearance

**Figure 4:**
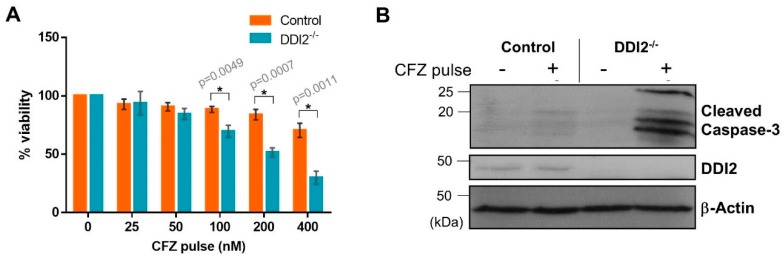
DDI2-deficient MDA-MB-231 cells exhibit greater sensitivity to carfilzomib in a pulse treatment scheme. (**A**) MDA-MB-231 control (wild-type) and DDI2^−/−^ cells were pulsed with increasing doses of CFZ (25–400 nM) for 1 h. CFZ was washed out with PBS and cells were given 24 h to recover in normal media, then analyzed by a luminescent cell viability assay. Viability percentage was determined via comparison to viability of untreated cells (100%). Error bars denote standard deviation (*n* = 3). (**B**) MDA-MB-231 control (wild-type) and DDI2^−/−^ cells were pulsed with 400 nM CFZ for 1 h. The CFZ was washed out with PBS and cells were given 24 h to recover in normal media, then analyzed by western blot using the antibodies indicated. β-Actin was used as a loading control. Blots shown are representative of three independent experiments. Significance (*p* value < 0.05) is denoted by asterisk.

Pharmacokinetic studies in clinical settings show that CFZ is rapidly cleared from the plasma after administration with a half-life shorter than 30 min [29]. To mimic this rapid clearance in vitro, we used an experimental design in which the cells were treated with CFZ for one hour, followed by wash-out of the drug. The cells were allowed to recover in normal media for 24 h, and then analyzed by cell viability assays. We found that the MDA-MB-231 DDI2^−/−^ cell line was more sensitive to CFZ-induced apoptosis when compared to control in both CellTiter-Glo (Figure 4A) and cleaved caspase-3 assays (Figure 4B). Thus, our results point to a clinically-relevant rationale for therapeutically targeting DDI2 in combination with CFZ treatment.

### 2.5. Protease-Dead DDI2 Acts in a Dominant Negative Fashion to Inhibit NRF1 Processing and Sensitize MDA-MB-231 Cells to CFZ-Induced Apoptosis

**Figure 5:**
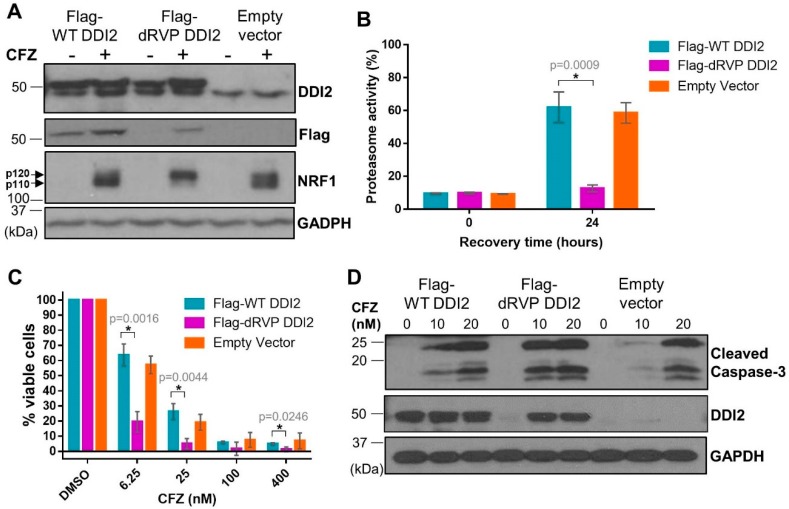
Protease-dead DDI2 acts in a dominant negative fashion to inhibit NRF1 processing and sensitize MDA-MB-231 cells to CFZ-induced cell death. (**A**) MDA-MB-231 cells stably expressing a wild-type DDI2 (Flag-WT DDI2), protease-dead DDI2 (Flag-dRVP DDI2), or an empty vector control construct were treated with 200 nM CFZ or equal volume DMSO for 4 h and analyzed by western blot using the antibodies indicated. GAPDH was used as a loading control. Blots shown are representative of three independent experiments. (**B**) MDA-MB-231 Flag-WT DDI2, Flag-dRVP DDI2, and empty vector cells were treated with 20 nM CFZ for an hour, and then the drug was washed out with PBS. The cells were allowed to recover for 24 h, after which the proteasome activity was measured and compared at the time points indicated. Error bars denote standard deviation (*n* = 3). (**C**) MDA-MB-231 Flag-WT DDI2, Flag-dRVP DDI2, and empty vector cells were treated with DMSO or increasing doses of CFZ (6.25-400 nM) for 24 h and analyzed by MTT assay. Viability percentage was determined via comparison to viability of DMSO-treated cells (100%). Error bars denote standard deviation (*n* = 3). (**D**) MDA-MB-231 Flag-WT DDI2, Flag-dRVP DDI2, and empty vector cells were treated with 10 or 20 nM CFZ or equal volume DMSO for 24 h, then analyzed by western blot using the antibodies indicated. GAPDH was used as a loading control. Blots shown are representative of two independent experiments. Significance (*p* value < 0.05) is denoted by asterisk.

DDI2 is similar to the well-characterized HIV-1 retroviral protease (RVP) in that both are aspartyl proteases [30,31]. Also similar to the HIV-1 RVP, DDI2′s only known protease substrate, NRF1, is cleaved between two hydrophobic residues [17,32]. Furthermore, like the HIV-1 RVP, DDI2 is predicted to homodimerize to become an active protease [30,31]. In the case of DDI2, homodimerization brings together the aspartic acid residues present in the catalytic triad (D[T/S]G) of the RVP domain of each monomer [33]. As there are no known chemical inhibitors for DDI2, we employed a genetic mutation (D252A) of the aspartic acid residue in the catalytic domain of DDI2 to create a protease-dead mutant (dRVP) for further characterization of the DDI2-NRF1 axis. Similar to DDI2 depletion, MDA-MB-231 cells expressing dRVP-DDI2 showed a profound defect in CFZ-induced NRF1 processing (Figure 5A), indicating that the protease-dead DDI2 acts in a dominant negative fashion to antagonize the endogenous DDI2. As a control, we observed that wild-type (WT) DDI2 and empty vector expressing cells demonstrated normal NRF1 processing in response to CFZ (Figure 5A). Interestingly, when compared to WT DDI2, the dRVP-DDI2 mutant seemed less stable and accumulated in response to CFZ (Figure 5A,D).

Next, to explore the downstream consequence of impaired DDI2 protease activity due to the dominant-negative effect of dRVP-DDI2, we performed a proteasome recovery assay. As previously described, we used 1 hour CFZ treatment to inhibit the proteasome activity to 90% of the original level, washed out the drug, then allowed the cells to recover. After 24 h, the WT-DDI2 and vector control expressing cells displayed a robust recovery in proteasome activity. The cells with dRVP-DDI2, however, showed a significantly crippled recovery (Figure 5B). Consequentially, the dRVP-DDI2-expressing cells were more sensitive to CFZ-induced apoptosis when compared to controls in MTT viability and cleaved caspase-3 assays (Figure 5C,D).

## 3. Discussion

NRF1-mediated proteasome bounce-back response is a well-established compensatory cellular mechanism to overcome proteasome inhibition and evade apoptosis [12,22]. Many proteins, including p97/VCP, NGLY1, and DDI2 [13,17,18,20], have been implicated in the activation of the transcription factor NRF1. Previous studies have suggested that DDI2-mediated proteolytic cleavage of the full-length p120 NRF1 releases the active p110 form from the ER membrane, allowing for migration to the nucleus, while the N-terminal stub harboring the transmembrane domain remains in the ER membrane [17]. However, based on our data presented here, we propose a refined model wherein NRF1 could be fully retrotranslocated into the cytosol in the full-length p120 form, where it could interact with and be cleaved by DDI2 into the p110 form. Regardless of cleavage by DDI2, we show that NRF1 may exist in the nucleus in its p120 or p110 form as an inactive or active transcription factor, respectively.

Previous studies have demonstrated that chemical inhibition of NGLY1 or p97—both of which are key players in the NRF1-mediated bounce-back response pathway—potentiated the apoptotic effect of proteasome inhibition in chronic myelogenous leukemia and cervical cancer cells [18] or multiple myeloma cells [24], respectively. In line with these findings, our results here show that DDI2-deficient MDA-MB-231 cells are more sensitive to CFZ-induced apoptosis. More importantly, this effect is recapitulated with a protease-dead DDI2 mutant as well. Taken together, these findings suggest that crippling the NRF1-mediated bounce-back response can both potentiate CFZ-induced apoptosis in hematologic cancers that already respond well to proteasome inhibition and may even sensitize previously non- or minimally-responsive tumors (e.g., solid tumors) to proteasome inhibition (reviewed in [7]).

Despite structural similarities to the HIV-1 protease, a previous study determined that HIV-1 protease inhibitors do not bind to the DDI2 protease domain, which may be due to the significantly larger substrate cavity possessed by DDI2 [30]. In our efforts to identify DDI2 protease inhibitors, we have explored multiple options ranging from protease inhibitor libraries to inhibitors predicted via structural analysis, albeit unsuccessfully (A.N; S.K.R. Chemical inhibitors for DDI2 protease. Unpublished; manuscript in preparation). HIV-1 protease inhibitors are known for severe toxicity [31], however a DDI2 protease inhibitor used in combination with proteasome inhibition would not require lifelong use and, hopefully, be better tolerated during short-term use.

DDI2 was recently identified to act as an oncogene to promote the tumorigenesis of colorectal cancer [34]. Though it was not demonstrated if the oncogenic property is related to DDI2′s protease activity or an alternative, such as serving as an ubiquitin receptor protein [35], it is possible that a DDI2 protease inhibitor may have anti-tumor activity independent of the NRF1-mediated bounce-back response. The Broad Institute’s Cancer Dependency Map Project revealed in their CRISPR and RNAi data sets that 21 of 29 breast cancer cell lines have a dependency score <0 for DDI2, indicating a pro-cancer role for DDI2 in these samples and furthering the hypothesis that inhibition of DDI2 as a single agent could have an anti-tumor effect in certain cancers. Regardless of the utility as a single agent, our results show that DDI2 depletion or protease domain mutation sensitizes MDA-MB-231 cells to proteasome inhibition, implying potential clinical utility of a combinational approach inhibiting DDI2 and the proteasome. Further research into the generalizability of this finding across breast cancer and other cancer cell lines and identification of a chemical inhibitor of DDI2 could enable the deployment of this combinational approach to potentiate proteasome inhibitor efficacy in already sensitive cancer types, and also potentially expand the repertoire of cancer types that can be treated with proteasome inhibitors.

## 4. Materials and Methods

### 4.1. Constructs

pQCXIP-6xHis-Flag-DDI2 (referred to as Flag-DDI2 in this paper), a construct expressing human DDI2 is a gift from Lan Huang (University of California Irvine, Irvine, CA, USA) and has been described previously [36]. We generated a vector control by removing the DDI2 coding sequence and re-ligating the plasmid. We also cloned murine DDI2 wild-type and protease dead-RVP (D252A mutation in the RVP domain) sequences into the above pQCXIP-6xHis-Flag vector. These are referred to as Flag-WT-DDI2 and Flag-dRVP-DDI2, respectively, in this paper.

The 20-mer CRISPR guide RNAs targeting coding sequences of mouse and human DDI2 (TGTTGTGATTCTACGACAGA for mouse and CATAAGAAGCCAATGATCTG for human) were cloned in to the lentiCRISPRv2 vector (gift from Feng Zhang, Broad Institute, Cambridge, MA, USA; Add gene plasmid # 52961 [25]). LentiCRISPR-EGFP-sgRNA1 was a gift from Feng Zhang (Broad Institute, Cambridge, MA, USA; Addgene plasmid # 51760).

Doxycycline-inducible shRNA expression construct targeting human DDI2 (referred to as inducible-shDDI2 in this paper) is a pTRIPZ construct purchased from Dharmacon/Horizon Discovery (Lafayette, CO, USA).

### 4.2. Cell Lines and Culture Conditions

NIH-3T3, MDA-MB-231 [37,38,39,40], and HEK293T cell lines and their derivatives were grown in Dulbecco’s modified Eagle’s medium (DMEM) supplemented with 10% fetal bovine serum (Atlanta Biologicals) and penicillin and streptomycin (Invitrogen) at 37 °C in a humidified incubator with 5% CO_2_.

For lentiviral and retroviral systems, viral particles were generated by transfection of HEK293T cells with the viral constructs and helper plasmids. Virus-containing medium was collected at 48 and 72 h post-transfection. Viral particles were precipitated using PEG-it (System Biosciences) and pelleted, then resuspended in serum-free medium and stored at –80 °C until use. Virus-containing medium, supplemented with polybrene, was used to infect the target cells in serum-free conditions. Puromycin (1 µg/mL) was used for the selection of all cell lines. The MDA-MB-231 DDI2^−/−^ cell lines infected with control or CRISPR-resistant Flag-WT-DDI2 expression constructs did not undergo selection.

Doxycycline at 1 µg/mL was used to drive expression of the inducible-shDDI2 for depleting DDI2. Cells were incubated with doxycycline for ≥72 h before experiments.

### 4.3. Western Blot Analysis

For preparing total cell lysates, cells were removed from culture plates via scraping, washed with cold PBS, and pelleted. Cell pellets were resuspended in RIPA lysis buffer (50mM Tris-HCl pH 8.0, 150 mM NaCl, 0.1% Triton X-100, 0.5% Sodium deoxycholate, 0.1% SDS) supplemented with protease and phosphatase inhibitor cocktail (Thermo Fisher Pierce, Waltham, MA, USA) and incubated on ice for 30 min, then centrifuged at 14,000 rpm for 20 min at 4 °C. Total protein was quantified using the Pierce BCA Protein Assay Kit (Thermo Scientific, Waltham, MA, USA), or the Bradford kit (Bio-Rad, Hercules, CA, USA), sample concentrations were equalized, and Laemmli sample buffer (Bio-Rad, Hercules, CA, USA) was added to 1×.

Alternatively, cell lysates were also prepared in sample buffer, where 2× Laemmli sample buffer (Bio-Rad, Hercules, CA, USA) diluted with RIPA was added directly to the cell culture plate followed by scraping and centrifugation.

For western blot, lysate samples were boiled for approximately 7 min. Proteins were separated by SDS-PAGE on 10%–15% acrylamide gels in SDS-PAGE buffer (250 mM tris, 1.92 M glycine, 1% SDS), then transferred to polyvinylidene difluoride membranes using the Trans-Blot Turbo Transfer System (Bio-Rad, Hercules, CA, USA) in transfer buffer (48 mM Tris, 39 mM glycine, 20% methanol). Membranes were blocked for 1 h with 5% non-fat dry milk powder in tris-buffered saline with tween (TBST [50 μM Tris-Base, 150 μM NaCl, 0.001 % Tween 20]), then incubated with primary antibodies overnight at 4 °C, washed three times with TBST, incubated with secondary antibody at room temperature for 1 h, and washed again before incubation with Pierce ECL Western Blotting Substrate (Thermo Scientific, Waltham, MA, USA) and film exposure or LICOR imaging. The antibodies used were specific for NRF1 (1:1000), Ubiquitin (1:1000), GAPDH (1:10,000), Lamin A/C (1:1000), Cleaved caspase-3 (1:1000) (all from Cell Signaling Technology, Danvers, MA, USA); DDI2 (1:1000, Abcam, Cambridge, MA, USA), Calnexin (1:10,000, Santa Cruz Biotechnology, Dallas, TX, USA), β-actin (1:10,000, Millipore Sigma, Burlington, MA, USA), and Flag (1:10,000, Millipore Sigma, Burlington, MA, USA). The secondary antibodies used were rabbit IgG HRP, and mouse IgG HRP (1:10,000; both from Bio-Rad, Hercules, CA, USA).

### 4.4. Quantitative Reverse Transcription PCR

RNeasy kit with DNase treatment (Qiagen, Germantown, MD, USA) was used to isolate RNA from frozen cell pellets. The iScript cDNA synthesis kit (Bio-Rad, Hercules, CA, USA) was used to convert 1 µg of RNA to cDNA. Quantitative reverse PCR (qPCR) was performed with iTaq universal SYBR green supermix (Bio-Rad, Hercules, CA, USA) in the C1000 Touch Thermal cycler (Bio-Rad, Hercules, CA, USA). Data was analyzed using CFX manager 3.1 (BioRad, Hercules, CA, USA). Levels of GAPDH expression were used for normalization. Statistics were determined by Student’s unpaired t-test. The forward and the reverse primers used for the qPCR reactions are as follows: PSMB4 (5′-TTC ACT GGC CAC TGG TTA TG-3′ and 5′-CGA ACG GGC ATC TCT GTA GT-3′), PSMB7 (5′-CTG TCT TGG AAG CGG ATT TC-3′ and 5′-GCA ACA ACC ATC CCT TCA GT-3′), PSMC4 (5′-TGG TCA TCG GTC AGT TCT TG-3′ and 5′-CGG TCG ATG GTA CTC AGG AT-3′), PSMD12 (5′-TCA CAG ACC TGC CAG TCA AG-3′ and 5′-AGG TTT TAG TCA GCC GAG CA-3′), and GAPDH (5′-AAC TTT GGC ATT GTG GAA GG-3′ and 5′-GGA TGC AGG GAT GAT GTT CT-3′).

### 4.5. Luciferase Assay

Luciferase assays were carried out using the NRF1-responsive 8×ARE-Luc construct described previously [22]. Cells were transfected with 8×ARE-Luc construct using Lipofectamine 3000 (Invitrogen/Thermo Fisher, Waltham, MA, USA). After 48 h, cells were treated with the indicated concentrations of CFZ overnight. Luciferase assays (Promega, Madison, WI, USA) were performed to measure firefly luciferase (under control of 8×ARE promoter) and renilla luciferase (under control of the human phosphoglycerate kinase promoter, which is ubiquitously expressed). Firefly luciferase expression was then normalized to renilla luciferase expression. Statistics were determined by Student’s unpaired *t*-test.

### 4.6. Proteasome Activity Recovery Assay

Cells were treated with the CFZ dose determined to inhibit proteasome activity by 90% for 1 h, then the CFZ was washed out with PBS and the cells were allowed recover in normal media. At the indicated time points after treatment, the cells were frozen in TE buffer (20 mM Tris pH 8.0, 5 mM EDTA) and stored at −80 °C. The assays were performed as described previously [12,22]. Briefly, cell lysates obtained by freeze-thaw lysis were mixed with Succinyl Leu-Leu-Val-Tyr-amino-4- methylcoumarin (Suc-LLVY-AMC) fluorogenic substrate to measure the chymotrypsin-like activity of the proteasome. Fluorescence was measured at 360/460 nm excitation/emission and the values were normalized to cell number as determined by CellTiter Glo (Promega, Madison, WI, USA). Statistics were determined by Student’s unpaired *t*-test.

### 4.7. Subcellular Fractionation

The Subcellular Protein Fractionation Kit for Cultured Cells (Thermo Scientific, Waltham, MA, USA) was used to isolate membrane-bound, cytosolic, and nuclear proteins in separate fractions. The protocol was followed per manufacturer’s instructions with added washes of the cell pellet with CEB after the cytosolic protein isolation and MEB after the membrane-bound protein isolation. After the MEB wash, 2× Laemmli sample buffer (Bio-Rad, Hercules, CA, USA) diluted with RIPA and supplemented with protease and phosphatase inhibitor cocktail (Thermo Fisher Pierce, Waltham, MA, USA) was used to lyse the remaining pellet containing the nuclear proteins. CEB and MEB were used at equal volumes, while the 1× sample buffer/RIPA was used at half of the CEB and MEB volumes. Calnexin, Lamin A/C, and GAPDH were used as fractionation controls for membrane-bound, nuclear, and cytosolic proteins, respectively, in the western blots.

### 4.8. Co-Immunoprecipitation Assay

MDA-MB-231 cells stably expressing flag-tagged DDI2 (flag-DDI2) or empty vector were treated with CFZ as indicated, pelleted, resuspended in IP buffer (50 mM Tris pH 7.4, 100 mM NaCl, 1 mM EDTA, 1% Triton X-100) supplemented with protease and phosphatase inhibitor cocktail (Thermo Fisher Pierce, Waltham, MA, USA), incubated on ice for 30 min, and then centrifuged at 14,000 rpm in a table-top centrifuge for 20 min at 4 °C. Pierce BCA Protein Assay Kit (Thermo Scientific, Waltham, MA, USA) was used to determine protein concentration. All samples concentrations were equalized and volumes adjusted to 250 µL, then 25 µL was set aside to run as the input (representative of total cell lysate). The remaining sample was used for immunoprecipitation with anti-FLAG Affinity Gel (Millipore Sigma, Burlington, MA, USA). The affinity gel (80 µL of the 50% slurry per sample) was blocked with 5% bovine serum albumin (BSA) in IP buffer for 1–2 h, pelleted, resuspended in IP buffer with half of the original volume (40 µL per sample), then added to the samples for overnight incubation in a tube rotator at 4 °C. The protein-bound affinity gel was then pelleted, washed with high salt IP buffer (300 mM NaCl) three times, then resuspended in 35 µL 2× Laemmli buffer diluted with IP buffer. Western blot protocol was then followed as described above.

### 4.9. Cell Viability Assays

MTT assay was used to measure cell viability. Briefly, cells were seeded at a density of 2 × 10^4^ in 100 µL of medium and allowed to attach overnight in 96 well plates. Cells were treated for 24 h with CFZ at the indicated concentrations in triplicate. After 24 h, 10 µL of 3-(4,5-dimethylthiazol-2-yl)-2,5-diphenyltetrazolium bromide (MTT) (5 mg/mL) was added to each well and incubated for 1 h, followed by PBS wash twice. Plates were dried and 100 µL of DMSO was added to each well to dissolve formazan crystals and absorbance was measured at 560 nm using GloMax Explorer microplate reader (Promega, Madison, WI, USA). Statistics were determined by Student’s unpaired *t*-test.
